# The Value of Pre- and Post-Stenting Fractional Flow Reserve for Predicting Mid-Term Stent Restenosis Following Percutaneous Coronary Intervention (PCI)

**DOI:** 10.5539/gjhs.v8n7p240

**Published:** 2015-12-16

**Authors:** Alireza Rai, Mostafa Bahremand, Mohammad Reza Saidi, Zahra Jalili, Nahid Salehi, Marzieeh Assareh, Gholamreza Amini Abarghoei, Hashem Kazerani

**Affiliations:** 1 Cardiology Department, Medical School, Kermanshah University of Medical Sciences, Kermanshah, Iran; 2 Psychiatry Department, Medical School, Alborz University of Medical Sciences, Karaj, Iran; 3 Kermanshah University of Medical Sciences, Kermanshah, Iran

**Keywords:** fractional flow reserve, percutaneous coronary intervention, restenosis, stent

## Abstract

Measuring fractional flow reserve (FFR) in percutaneous coronary intervention (PCI) has predictive value for PCI outcome. We decided to examine the utility of pre- and post-stenting FFR as a predictor of 6-month stent restenosis as well as MACE (major adverse cardiac events). Pre- and post-stenting FFR values were measured for 60 PCI patients. Within 6 months after stenting, all patients were followed for assessment of cardiac MACE including myocardial infarction, unstable angina, or positive exercise test. Stent restenosis was also assessed. Cut-off values for pre- and post-stenting FFR measurements were considered respectively as 0.65 and 0.92. Stent restenosis was detected in 4 patients (6.6%). All 4 patients (100%) with restenosis had pre-stenting FFR of < 0.65, while only 26 of 56 patients without restenosis (46.4%) had pre-stenting FFR value of < 0.65 (P = 0.039). Mean pre-stenting FFR in patients with restenosis was significantly lower than in those without restenosis (0.25 ± 0.01 vs. 0.53 ± 0.03, P = 0.022). Although stent restenosis was higher in patients with post-stenting FFR of < 0.92 (2 cases, 9.5%) than in those with FFR value of ≥ 0.92 (2 cases, 5.1%), the difference was not statistically (P = 0.510). Pre-stenting FFR, the use of longer stents, and history of diabetes mellitus can predict stent restenosis, but the value of post-stenting FFR for predicting restenosis was not explicit.

## 1. Introduction

Fractional flow reserve (FFR) is a useful technique used in coronary catheterization to measure pressure differences across a steno tic segment in a coronary artery (Pijls et al., 1996). FFR is basically defined as the pressure distal to a stenosis in a coronary vessel relative to the pressure before the stenosis. Thus, it can express the maximal flow down a vessel in the presence of a stenosis compared to the maximal flow in the hypothetical absence of the stenosis (Tonino et al., 2009). In fact, the measurement of this indicator directly depends on both lesion morphology and distal myocardial bed requirements, but it is independent to hemodynamic changes such as heart rate, blood pressure or myocardial contractility (Pijls et al., 1995). Physiologically, the value of less than 1 indicates abnormal FFR (de Bruyne, 1996); however in some clinical studies, a cutoff value < 0.75 has been considered for assessing inducible myocardial ischemia, with a sensitivity of 88%, specificity of 100%, and predictive accuracy of 93% (Pijls et al., 1996).

Considering the high diagnostic accuracy of FFR in clinical setting, this indicator has been used for evaluating early and late outcome of coronary revascularization in some clinical trials. Some non-randomized studies suggested that patients who were treated based on FFR guidance had better outcome compared to those treated based on angiographic appearance alone (Wongpraparut et al., 2005; Legalery et al., 2005).

For example, in the DEFER study, FFR was used as an index to determine the need for stenting in patients with intermediate single vessel disease. Patients with a FFR of less than 0.75 had a significantly worse long-term outcome. However, patients with FFR ≥ 0.75 had five time slower rates of cardiac death and acute myocardial infarction as well as higher improvement in anginal status with PCI compared with those with lower FFRover 5-year follow-up (Pijls et al., 2007). In another study to compare the FFR versus angiography for multi-vessel evaluation (FAME study) and at two-year follow-up time, FFR-guided patients had significantly lower major adverse cardiac events (MACE) including death, non-fatal myocardial infarction, and need for repeat revascularization (Pijls et al., 2010).

Although the value of FFR for discriminating favorable and poor outcome following coronary revascularization has been clearly determined, its value for predicting stent restenosis after PCI procedure remains unclear. Although some clinical predictors have been identified to predict stent restenosis such as diabetes mellitus, long stent length, small reference diameter, and final percent diameter stenosis (Lemos et al., 2004; Kastrati et al., 2006), the predictive power of these indices has been estimated low and thus using a blood flow rate-based system is preferred to assess stent restenosis. Thus, the study was designed to examine the utility of pre- and post-stenting FFRvalues as a predictor of stent restenosis. In addition to stent restenosis, 6-month MACE was also evaluated.

## 2. Materials and Methods

A total of 60 consecutive patients who underwent PCI (percutaneous coronary intervention) at our center were included in this cohort study. Exclusion criteria were factors that could either impair the correct measurement of FFR such as severe left main coronary artery stenosis, prior bypass surgery, visible collateral development, complete occlusions of the target vessel, and side branch involvement or ethical considerations that an FFR measurement in a special condition could be potentially dangerous for the patient including severe left main coronary artery stenosis, acute coronary syndromes, urgent coronary revascularization, or recent myocardial infarction. Before stenting, FFR was measured in all participants by determining Pd/Pa ratio, where Pd represents mean coronary pressure distal to the steno tic segment measured by the pressure wire, and Pa represents mean aortic pressure measured by the guiding catheter. The measured FFRs were categorized as < 0.65 and ≥ 0.65. Once more after stenting, FFR was measured with dividing patients into FFR of 0.85-0.92 or ≥ 0.92.

Within 6 months after stenting, all patients were followed for assessment of occurring cardiac events including myocardial infarction or unstable angina. The patients with one of these cardiac events underwent coronary angiography and those with a FFR < 0.75were categorized as stent restenosis group. Other patients without cardiac events also underwent exercise tolerance test (ETT) and those with positive ETT results and concomitant FFR of < 0.75 were considered as stent restenosis. However, those with positive ETT and FFR of ≥ 0.75 were considered as non-restenosis.

We compared categorized variables between restenosis and non-restenosis groups using the Chi-square test or Fisher’s exact test, if required. P values of 0.05 or less were considered statistically significant. All the statistical analyses were performed using the SPSS software version 16.0 (SPSS Inc., Chicago, IL, USA).

The study protocol was approved by the Ethics Committee of our university.

## 3. Results

Mean (±SD) age of the patients was 58.0 (±4.7) years (range, 43 to 75 years). Regarding underlying risk profile, 23 patients (38.3%) were diabetics and 24 (40.0%) had high LDL levels. In this study, 60 stents were used (29% metal stent and 71% drug-eluting stent) with a mean length of 7.22 mm. Thirty-one patients had a stent shorter than 20 mm, while the length of stent in 5 patients was longer than 35 mm.

Stent restenosis was detected in 4 patients (all had drug-eluting stents, 6.6%) at 6-month follow-up.

In total, mean (±SD) pre- and post-stenting FFR values were 0.51 (±0.28) and 0.93 (± 0.04), respectively. All four patients (100%) with stent restenosis had pre-stenting FFR values of lower than 0.65, while only 26 of 56 patients without restenosis (46.4%) had FFR of lower than 0.65 (P= 0.039). Also, mean (±SD) pre-stenting FFR in patients with restenosis was significantly lower than in those without restenosis (0.25 ± 0.01 vs. 0.53 ± 0.03, P= 0.022).

Post-stenting FFR in all subjects were higher than 0.85; 21 patients (35%) had FFR of 0.85-0.92 and 39 cases (65%) had FFR of 0.92-1. Although stent restenosis was higher in patients with post-stenting FFR of < 0.92 (2 cases, 9.5%) than in those with FFR value of ≥ 0.92 (2 cases, 5.1%), the difference was not statistically (P = 0.510).

There was a significant association between the rate of stenosis and length of stent (P < 0.001); of four patients with stent restenosis, two patients had stents of 30-35 mm and other 2 patients had stents longer than 35 mm, whereas only 3 non-steno tic stents were longer than 35 mm. As shown in Table 1, longer length of stent and history of diabetes mellitus were associated with higher rate of stent restenosis.

**Table 1:** Relationship between baseline comorbidities and occurrence of stent restenosis

	With restenosis (N = 4)	Without restenosis (N = 56)	P-value
**High LDL**	2 (50.0%)	22 (39.0%)	0.677
**Diabetes mellitus**	4 (100%)	19 (33.0%)	0.009
**Hypertension**	2 (50.0%)	25 (44.0%)	0.831
**Current smoking**	2 (50.0%)	22 (39.0%)	0.677
**Stent length > 30 mm**	4 (100%)	8 (14.3%)	< 0.001

The overall 6-month MACCE rate was 10.0%; 2 patients experienced non-fatal STEMI, 2 had unstable angina, and 2 had positive ETT. No significant associations were found between the measurement of post-stenting FFR and any of these cardiac events (Table 2).

**Table 2:** Relationship between post-stenting FFR and mid-term cardiac events

	FFR < 0.92 (N = 21)	FFR ≥ 0.92 (N = 39)	P value
**ST-elevation MI**	2 (9.5%)	0	0.134
**Unstable angina**	0	2 (5.1%)	0.545
**Positive exercise tolerance test**	0	2 (5.1%)	0.545

## 4. Discussion

The present study attempted to assess the predictive value of both pre- and post-stenting FFR for predicting restenosis as well as to study the value of this parameter for predicting mid-term MACE in these patients. As previously pointed, the certain role of this cardiac index for assessment of poor outcome of revascularization by PCIhas been demonstrated, however there is limited evidence regarding its usefulness for determining restenosis. Based on our study results, although pre-PCI measure of FFR could predict mid-term restenosis, but post-stenting FFR did not have such predictability. In a similar study by Ruscazio and colleagues, low FFR detected one month after PCI in the left anterior descending coronary artery had the potential to identify patients at higher risk for developing coronary restenosis indicating the need for further clinical assessment (Ruscazio et al., 2012). In another study by Ishii et al., patients suffered from restenosis had a lower FFR at post stent deployment, suggesting the decreased FFR might be a useful predictor for stenting restenosis (Ishii et al., 2011). Krüger et al. also showed that FFR measurement was an applicable tool for decision making in patients with stent restenosis so because the threshold of stenosis diameter of coronary lesions for pathologic FFR measurement defined as FFR < 0.75 was similar for stent restenosis and native coronary stenosis (Kruger et al., 2005). Besides, similar to our study, Lopez-Palop et al. showed a poor correlation between angiographic quantification and FFR of moderate in-stent restenosis. In their study, an FFR value of > 0.75 was obtained in 63% of lesions that half of them were accompanied with stenosis > 50% (Lopez-Plaop et al., 2004). Our insignificant observation on low predictive power of post-FFR for stent restenosis might be due to the presence of some underlying confounders affecting coronary artery flow rate such as diabetes or hypertension. It also might be related to different cutoff points of FFR for defining stenosis or even could be influenced by small sample size or short-term following of the patients. As shown by Amaya and colleague, post-stenting FFR can be very useful for prediction of restenosis in patients without diabetes mellitus, whereas it may not be an appropriate tool in patients with diabetes mellitus that it might be attributable to target lesion revascularization rather than FFR (Amaya, Takazawa, Tanaka, & Yamashina, 2002). On the other hand, the higher prevalence of hypertension and diabetes in patients with native coronary stenosis can possibly serve as a confounding factor, because it is associated with a higher microvascular resistance, potentially leading to a higher FFR value (Meuwissen et al., 2001).

In another attempt, we revealed that the main predicting factors for stent restenosis included longer length of stent and history of diabetes mellitus. In previous studies, main predictors of restenosis were considered to be diabetes mellitus (Kornowski et al., 1997; Ari et al., 2010), smaller reference diameter (Elezi et al., 1998), diffuse and long lesions (Kobayashi et al., 1999), and minimum stent area (Kasaoka et al., 1998; Morino et al., 2001). On the other hand, small diameter and area can concomitantly predict restenosis in order to potential changes in coronary flow resistance rate and higher susceptibility to atherosclerotic lesion in these conditions.

In conclusion, pre-stenting FFR, the use of longer stents, and history of diabetes mellitus can predict stent restenosis, but the value of post-stenting FFR for predicting restenosis has been already ambiguous and needs further larger trials.
